# Edinburgh Medico-Chirurgical Transactions

**Published:** 1824-09-01

**Authors:** 


*Transactions of the Medico-chirurgical Society of Edinburgh.* Vol. I. Edinburgh, 1824.

[Second Analytical Article.]

### Art. I.

*Appearances observed in the Dissection of two of three Individuals presumed to have perished in the Storm of the* 3d *of November*, 1821, *and whose Bodies were discovered in the Vicinity of Leith, on the Morning of the* 4*th of the same Month;—with some Reflections on the Pathology of the Brain.* By George Kellie, M. D. &c.

Saturday night, the 3rd of November, 1821, was remarkably cold, tempestuous, and dark;—next morning three individuals were found dead, and extended on the ground, in the vicinity of Leith. One of the three was recognized by his friends, and removed:—the others became the subjects of medico-legal examination. Drs. Cheyne and Kellie inspected the bodies. One was a middle-aged man, perhaps 40—the other an elderly female. The external appearances of the man presented nothing remarkable. The body generally might be described as presenting more than usual freshness and soundness. When the scalp was divided, very little blood flowed from the integuments. The dura mater was somewhat congested, suffused, and heightened in colour. Its sinuses were loaded with dark blood. Veins of the pia mater were turgid and injected. Milky serous effusion between the pia mater and arachnoid. Cerebrum, in texture and colour, sound. Between three and four ounces of serum in the ventricles, and at the base of the brain. *Abdomen.* Small intestines very deeply coloured— especially the ilium, which was red, and presented a fine specimen of vascular congestion. No appearances of this kind in the stomach or colon, the former of which was nearly empty; its mucous membrane presenting a few spots of a coffee-colour. Liver congested. No other morbid appearances in the abdominal viscera.

The female appeared beyond the age of sixty. No blemish, or external injury. The veins of the dura mater were injected, and its sinuses loaded—vessels of the pia mater congested—three ounces of serous fluid in the ventricles, and at the basis cranii. On raising the omentum, "the small intestines exhibited precisely the same appearances as in the man; the same redness, not in patches, but over the whole extent of the bowel, and occasioned by the same general and minute injection of the vessels, profusely ramified beneath the peritoneal coat." The stomach and colon, in this case also, were of the usual pale colour—the former containing a few ounces of viscous fluid, and some fragments of undigested beef. There were a few purplish spots on the mucous membrane of the stomach. No other unusual appearances.

*Medico-legal Report.* Our authors stated their conviction that the above unfortunate victims had not fallen in consequence of any violence or external injury—but that they perished from the severity of the weather.

During the said night, there was a furious gale from the north-east, accompanied by rain, sleet, and snow— circumstances that would greatly augment the benumbing influence of the cold (which, in itself, was not below the freezing point) if the individuals, benighted, fatigued, and worn out, gave up exertion, in despair of finding their way, and lay down to sleep—alas, to wake no more!

Some traces were ascertained of the history of these individuals; and it appeared probable that all three had lost their way, and were exposed to the dire effects of the storm, from an early hour in the evening. Being Saturday night, when the lower classes receive their wages, and when there is a kind of periodical propensity to indulgence in spirituous potations, it is not impossible that these poor creatures might have been, in some degree, intoxicated—but of this there is no proof,*

Our author has not been successful in finding more than one recorded dissection of persons dying from the effects of cold. We have also tried back, and with the same want of success. We apprehend, however, that there must be some cases on record, though we have not been able to find them. The case quoted by Dr. Kellie, is from the sixth volume of Haller's Disputations—and in it "*the vessels of the brain were observed turgid with blood, and in the ventricles was found an effusion of serous lymph.*" The *physiological* effects, however, of a very low temperature on the human frame, are well known, and have been repeatedly described, from Galen down to Bankes, Humboldt, and Parry. They bear a striking resemblance to the symptoms observed in the order comata. Cullen, considering cold as one of the causes which produce apoplexy, does not suppose that it does so by *compression*, but by destroying the mobility of the nervous power. In the three cases above alluded to, there are pretty evident proofs that compression must have existed. Congestion of vessels, and effusion of fluids, could not easily take place independent of compression—or rather, of *pressure.* Here, our author enters into a consideration of the peculiarity of the circulation within the head. It is evident that, as the brain is enclosed within a long case, and in itself nearly incompressible, there can only be the same, or very nearly the same quantity of fluids at all times within the cranium. Thus, if the arterial system be surcharged, the venous must be proportionally depleted—if an effusion of six or eight ounces of serum or blood take place, there must be a corresponding quantity of blood forced out of the head. All this, we think, is unquestionable, and required not the algebraic proofs which Dr. Kellie has brought forward. But compression is one thing, and *pressure* is another—at least, there is no necessity that the *former* should exist where the *latter* obtains. Allowing the brain to be completely incompressible (which it is not) a strong action, and full state of the general vascular system, must cause more *pressure* on it than a weak and depleted condition of the heart and arteries:—and thus it is that we relieve the head from *pressure*, without perhaps altering, in any appreciable degree,

---

* Although vinous and spirituous potations appear to have the effect of enabling us to resist the impressions of cold, at least, for a time, yet, as they greatly augment the tendency to somnolency, they would, under the circumstances above-detailed, contribute to the fatal event.—Rev.

the actual quantum of blood circulating through its vessels. But there is another point of view in which we should regard vascular turgescence and effusions. On opening the heads of those who have died with symptoms of compression, or pressure, we generally, if not always, find certain parts of the vascular system of the brain more distended than others. Here then we have *unequal pressure* on the encephalon, which inequality of pressure, indeed, is pretty well indicated by the inequality of functional disorder manifested before death. In cases of extravasation of blood or other fluids into the natural cavities, or into lacerations of the brain, must not this local, unequal, or partial pressure be still more operative, even if there be not a drop more of blood at this than at any other, within the cranium?

Here Dr. Kellie has criticised Dr. Abercrombie's doctrine, on this point, in almost the very words we used at p. 10, 11, and 12 of the first number of our Analytical Series, for June, 1820. Dr. Kellie has made a considerable number of experiments on animals which he bled to death, or poisoned, with a view of ascertaining whether, and how far, the vessels of the brain could be depleted by detractions of blood *usque ad mortem.* The result of these experiments may be thus stated.

"That though we cannot, by any means of general depletion, entirely or nearly empty the vascular system of the brain, as we can the vessels of the other parts of the body, it is yet possible, by profuse hæmorrhages, to drain it of a sensible portion of its red blood;—that the place of this spoliation seems to be supplied both by extra and intravascular serum, and that watery effusion within the head is a pretty constant concomitant or consequence of great sanguineous depletion.

"If, instead of bleeding, as in our examples, 'usque ad mortem,' we were to bleed animals more sparingly and repeatedly, I have no doubt that we should succeed in draining the brain of a much larger quantity of its red blood; but in such experiments, we should, I think, find a larger effusion of serum, and be satisfied that many vessels, destined to circulate red blood, were filled with serum only, and even the larger trunks with a very thin and diluted blood." 116.

So in a case of exquisitely marked anemia, examined by Dr. Combe and himself, although very little blood was found in the vessels of the brain, or its membranes, "yet they contained more than the usual relative quantity of fluid which had circulated during life; a pale and colourless blood, it is true, but in such quantity within the head that, had it been less serous—more highly coloured—more, in short, like true blood—the vascular system of this brain would have presented little more striking or remarkable to the eye of the dissector, than a somewhat less than usual turgescence, perhaps, of the sinuses and larger vessels, and a profusion of effused and interstitial serum.* This brings our author to the second part of his paper—" Reflections on the Pathology of the Brain."

Having shewn that there are natural obstacles to the free depletion of the brain, not existing in any other part of the body—that we cannot, in fact, materially lessen the quantum of blood within the cranium, by profuse venesection, without leaving an equivalent to this spoliation in an increase of serous effusion, serving to maintain the plenitude of the cranium, it seemed reasonable to our author, that, by removing a portion of the skull, the defence from the pressure of the atmosphere would be taken off, and we should thus succeed in producing a much greater depletion of the cerebral vessels, by general blood-letting, than could otherwise be effected. In order to prove this, three dogs were trepanned, and then bled to death. The brain was depressed, or shrunk in at the place of perforation of the skull, and the vessels internally were found depleted.

We may just remark that, whatever might be the result of experiments of this kind on animals, they would be perfectly impracticable on man—and for this reason, that, unless the dura mater was detached from the skull, to which it so strongly adheres, no action of any consequence would be produced by the removal of a circular piece of bone.

The same causes which prevent depletion of the cerebral vessels, ought also to prevent over repletion. The subject of hanging and drowning is brought forward by our author, in illustration. It is evident that the rope prevents the return of blood from the head by the veins, and cannot prevent the transmission of blood along the vertebral arteries, at least, if it could gain admission into the cerebral vessels, after the suspension commences. Dissection, in such cases, does not shew the cerebral vessels particularly injected; nor, indeed, do we think this injection could be fairly expected. The interruption to the venous return of blood, lasts only a few minutes—the flow of blood along the carotids, must be nearly, if not entirely stopped by the ligature—the vessels of the brain are healthy—and finally, what might be injected into the vessels of the brain, by the vertebral arteries, during suspension, may be very fairly supposed to recoil from the vessels, when the ligature is taken off, and before the head is opened.† Dr. Kellie offers a curious argument against this conclusion—namely, that as " the cord is

* See the details of this case, in the next paper, analysed.

† The same remark we find to be made by Morgagni, Epis. XVIII, II.

never removed till the subject is cut down, and laid in the horizontal posture, the blood can have no tendency to gravitate towards the heart." What, are we to count for nothing the elasticity of the arteries, if over-distended?—How does Dr. Kellie account for the rush of the blood from the vessels at the base of the skull, when the brain was removed, as stated by himself, at the bottom of page 133?—besides, we are informed by Dr. Kellie, who himself examined the necks of people who were hanged, that the noose slips up on one side of the neck, behind the ear, "and there is, consequently, a space on this side, corresponding to the rising of the noose, *which is not embraced by the cord*, and where the veins, returning the blood from the head, *are subjected to little, if any, pressure*." If this be the case, how should we expect to find congestion of the cerebral vessels, knowing, as we do, the free communication between the sinuses of the brain, and the readiness with which the blood would be returned by one of the internal jugulars.

Our experience does not coincide with that of Dr. Kellie, on the following point.

"Some diseases of the heart, and of its larger vessels, constitute cases in which we might expect to find the brain more plethoric and congested than usual. In obstruction of the auriculo-ventricular valves, an obstacle exists to the free return of the venous blood from the head; and in muscular enlargement of the heart, not unfrequently complicated with contraction of the descending aorta, the impetus of the blood upon the brain is often powerfully increased. Sometimes, too, these conditions either of which seem so well calculated to produce fullness end congestion of the vessels within the head, are found united in the same case, obstruction, viz. to the return of venous, and increased impetus of arterial blood. Yet, I believe it will be found that, in a sound condition of the brain and its vessels, such diseases of the heart have little or no tendency to produce lethargy, palsy, or apoplexy, nor by consequence plethora, congestion, or disordered circulation within the head, although the livid, bloated, and sometimes swollen countenance, and the turgid and throbbing neck, bear ample testimony to the existence of plethora, obstruction, and congestion, in the vessels exterior to the cranium. Of the several cases of enlargement, and of other structural diseases of the heart, which have come under my own observation, not one of the patients had lethargic or apoplectic symptoms. One only had a partial paralytic affection of the right arm." 141.

We have seen several cases where there was every reason to ascribe the affection of the head to the hypertrophy of the heart—and our readers know, that this connexion is very universally admitted by Continental writers.* Another phenom-

* Baglivi was, we believe, the first to notice this connexion, when relating the case of the celebrated Malpighi—then Gibellini, and Lieutaud. Richenon, which almost every practitioner must have observed, is the irritability and irascibility of patients with active aneurism of the heart, and consequently, great impulsion of blood on the brain. Indeed, the case brought forward by Dr. Kellie, in corroboration of his own doctrine, militates, in our opinion, against it. A gentleman, who had been for some time ailing, complained to Dr. Kellie of inability of forming the letters in writing, which disability was accompanied by "head-ache and vertigo," relieved by bleeding, and other evacuations. Soon after this, decided symptoms of enlargement of the heart, and aorta came on, which ended fatally in a short time. The heart was found to be double its natural size, as was also the arch of the aorta. The head was not examined.

In another case of enlarged heart, our author notes the following phenomenon.

"On looking upwards, to the whitened ceiling of a room, he saw a darkened spectrum, which vanished and reappeared with great regularity. It was soon discovered that the appearance of this umbra was synchronous with the systole of the heart, so that he used often, in my presence, to count his pulse with the utmost precision, by keeping his eye fixed on the ceiling, and numbering every appearance of the spectrum." 144.

It is very true that the greater number of organic diseases of the heart will come to a fatal termination before they occasion such disease in the vessels of the brain, as to lead to apoplexy. And, again, there are many diseases of the heart, caused by puckering or narrowing of the root of the aorta, in which case, there would be no violent propulsion towards the head, but the reverse.

Considering that Dr. Kellie acknowledges that *pressure* may be made on the brain, by general plethora, or excessive action of the heart, although there may be no increase of blood in its vessels, we were, certainly, a little surprised, at the following passage, in page 147 of the volume under review.

"Whatever tendency such diseases of the heart, and larger vessels, may have to produce *plethora, congestion, or deranged circulation within the head*, that tendency is opposed and counteracted by the physical

---

erand distinctly traced apoplexy to aneurism of the heart, as may be seen in the third volume of his Nosographie Chirurgicale. Legallois has related a remarkable instance of this kind, and still more recently, Richerand has detailed the case of the celebrated Cabanis, who died of apoplexy, while labouring under an enormous hypertrophy of the left ventricle of the heart. But, it is to M. Bricheteau we owe the fullest investigation of this subject. He has brought forward a body of evidence, from clinical observations at the Hotel Dieu, in proof of the connexion between cardiac aneurism and apoplexy, which Dr. Kellie's partial and scanty *negative* evidence can have but little power to invalidate. *See Journal Complementaire, for July* 1819—Rev.

situation of the brain, and the peculiar confinement of its vascular system." 147.

Dr. Kellie endeavours to explain away the connexion between cardiac and cerebral affections, on the principle of *coincidence*, not consequence, and he has made out a very good case, for a discussion in a medical society, but not such a one as shall produce conviction in the mind of the attentive observer. Indeed, when we call to mind the various cases of cardiac disease which have passed under our eye, and recollect their almost invariable attendant—*mental irritability*, we cannot permit the sophistry of *coincidence* to blind us against the host of facts which prove, as far as medical proofs can ever go, the intimate connexion between organic affections of the heart and disturbance of function, at least, in the brain. That long-continued derangement of function generally ends in alteration of structure, no practical physician can now entertain a doubt; and that this is especially the case with the brain, we are convinced from experience.

Dr. Kellie, at page 149, brings forward a case which, he thinks, proves to a demonstration, the entire independence of the two classes of disease. We shall first present a faithful abstract of the case.

A man, *thirty-two years of age*, was admitted into a public institution, on the 9th of January 1823, for the treatment of amaurosis. He had pain in the head, for six years previously, "*though in other respects his general health appeared good.*" On the seventh day after admission, (and apparent good health) he complained of dyspnœa, cough, sickness, thirst, and had quick pulse. On the 9th day, he became comatose, and on the 10th from his entrance, he died.

*Dissection.* Vessels of the brain not particularly distended;—effusion under the arachnoid membrane— brain itself particularly firm throughout—no fluid in the ventricles—the vessels at the basis of the brain were diseased, as the vertebrals, basilar, Circulus Wilisii, being thickened, their coats extremely tough, and some of them studded with spots of ossification. The heart was much enlarged, the ventricles, especially the left, being in a state of active hypertrophy. There was a large polypus in the right auricle, passing into the cavæ.

On an impartial review of the above case, we can see nothing to warrant the presumption that the two diseases were so completely independent and unconnected as Dr. Kellie makes them to be. On the contrary, we should draw a conclusion in favour of their connexion. Is it usual to find the cerebral vessels of a young man of 32, tough, thickened, and ossified?—Certainly not. But the circumstance of active hypertropy of the heart co-existing—and (for aught that is shewn to the contrary) preexisting, comes in as an explanation of so early a development of cerebral disease, which otherwise would be very difficult of solution. We perfectly agree with our author, and it is well known we have always enforced the doctrine, that the immediate cause of apoplexy must be sought, in a diseased state or condition of the cerebral vessels themselves. But then, this diseased condition may be occasioned or accelerated by such a disease, as deranges the circulation of the blood in the head. How frequently do we find the function and structure of the lungs affected by organic diseases of the heart? How often effusions into the various cavities?—Why then should the head be considered as exempt from participation in the disorder of an organ, that exercises such a powerful influence over the whole system, as the heart?

Our author argues that, if, in such a condition of cerebral vessels, as detailed above, the enlarged heart was not capable of producing apoplexy, it is not likely it would do so under circumstances of a healthy state of the said vessels. But, surely, this is loose reasoning. Did not the patient, after all, die of apoplectic affection?—There is no evidence of his dying of the cardiac disease. The outward phenomena of this disease were so slight, that Dr. Kellie did not know there was any thing of the kind existing, till the dissector disclosed it. The man, therefore, died of the disease in the head, and there is every probability that the said disease was accelerated, if not caused by the hypertrophy of the heart. In this case, then, we maintain that there is nothing against, and much in favour of, the connexion of which we are treating.

The remainder of Dr. Kellie's paper is taken up with examples of obstruction to the return of blood from the head, by ligatures, tumours, &c. &c. not followed by compression of the brain; but of these instances we need not speak. The resources of nature are, no doubt, wonderful in compensating for, or obviating, the loss of parts, and the various accidents to which flesh is heir; and if all the remarkable instances of this kind were collected, and arranged on one side, we should have a picture that would represent nature as perfectly infallible, and as no longer requiring the least assistance from art. But when we look at the other side of the scene, and contemplate the host of trifling deviations that baffle both Nature and the physician, we shall be induced to lower our admiration a little of the Vis Medicatrix Naturæ. The whole paper tends to prove what no one, we believe, will deny, that, from the peculiarity of the circulation in the head, little alteration in the *quantum* of fluids permeating its vessels, can be produced by art—

but that the *pressure* of such fluids on the brain may be materially diminished by lessening the quantum of blood and other fluids in the general system, and diminishing the force of the heart and arteries. These positions we have, ourselves, long maintained in various parts of this journal, and claim for them no praise as to novelty, for they were long ago maintained by Monro—and probably by many others before him.

"For," observes Monro, "as the substance of the brain, like that of other solids of our body, is *nearly incompressible*, the quantity of blood within the head must be the same, or very nearly the same, at all times, whether in health or disease, in life, or after death, those cases only excepted in which water or other matter is effused or secreted, from the blood vessels; for in these, a quantity of blood, equal in bulk to the effused matter, will be pressed out of the cranium."—*On the Brain.*

And again, the same author remarks—"the less compressible we suppose the substance of the brain to be, the more readily we understand how the whole of it may be affected by a plethora, or increased momentum of the blood." *Ib.*

Much has been said on the incompressibility of the brain; but we do not know that it has ever been proved. When we reflect on the differences which are presented in different brains—some of them exhibiting the vascular system, arterial and venous, injected, as if with red and blue wax—while the same system, in other brains, appears nearly exanguious, we cannot but conclude that considerably more blood is contained in the one class than in the other. Granting that the substantia cerebri is incompressible, or nearly so, can the same be said of the coats of the vessels, and the cellular substance which surrounds them wherever they penetrate?—We think not. And if not, here is a field for an increased quantum of blood within the cranium, under certain circumstances of plethora, great vascular action, or a debilitated state of the cerebral vessels themselves. In this way, we can conceive that very considerable additional pressure may be made on the brain, to the great derangement of its functions, and ultimate alteration of its structure.

Finally, we may be permitted to express our opinion that Dr. Kellie's experiments and observations have added very little indeed to our previously existing stock of knowledge respecting the *pathology* of the brain—nor is it calculated to make any alteration in the *treatment* of cerebral affections. We are ready to agree, however, with the very able and intelligent author, that the causes which have been enumerated by writers, as tending to produce congestions of blood in the head, have been over-rated—that Nature has guarded against repletion and depletion of the brain (as much as she could)—that while the structure of this organ remains *healthy* and *unchanged,* and its *vessels sound,* those causes are little capable of occasioning plethora, congestions, effusions or comatose diseases—and, finally, (as indeed we have repeatedly observed ourselves) the great causes of apoplexy, are changes which take place in the brain itself, its vessels, or its membranes.

### Art. II.

*Case of Anæmia.** By. J. S. Combe, M.D. &c. &c. &c.

This is the case alluded to by Dr. Kellie, in the paper we have just reviewed. There is something so mysterious and ghostly about this disease, that, in a more superstitious age, patients labouring under it might readily be supposed the victims of some blood-sucking vampyre.

This disease has been described, or alluded to by various Continental writers, but, as far as we know, Lieutaud was the first (Medecine Pratique) to give an exact description of it. In the Journal de Medecine, par Corvisart, Leroux et Boyer, vol. 1, there is a very full account, by professor Hallé, of Anæmia, as it affected epidemically, (or rather endemically) the workmen in a coal pit, near Valenciennes. The disease affected the men of one gallery or pit *only*, and it assailed each successive reinforcement of workmen employed there. The gallery was at length shut up—but all who had ever been in it suffered from the disease, although some of them were not seized till three or four months after the closure of the gallery. The air of this coal mine (which was 120 feet below the surface of the earth) was strongly impregnated with sulphuretted hydrogen gas, and also carbonic acid.

The disease commenced with severe colicky pains in the stomach and bowels, tightness in the chest, palpitation, prostration of strength, black alvine evacuations. After ten or twelve days of these symptoms, the pains ceased, the pulse became feeble and quick, skin pale and sallow, debility excessive, profuse sweats. This condition lasted several months, and, in some instances, a whole year. Finally, the first symptoms, in many cases, returned, the abdominal pain being dreadful, with purulent dejections, and sudden death. Four of these patients were brought to Paris, where one of them died. On dissection, the vessels were found almost entirely destitute of blood, and the viscera pale and flaccid. The same was the case in all the other dissections that were made. The only medicines that proved useful, were bark, steel, and nourishing diet.

* *a privative,* and *αἷμα blood.*

*Dr. Combe's Case.* In the month of July, 1821, our author was consulted by Alexander Haynes, who exactly resembled a person just emerging from a state of syncope—his face, lips, and whole cutaneous surface being of a deadly pale colour, while the albuginea of the eye was blueish. His motions and speech were languid—respiration hurried on exertion—pulse 80, and feeble—tongue dry and furred—lips and fauces pale—bowels irregular, generally relaxed—stools dark and fetid—urine pale and copious—appetite impaired—stomach rejects food—constant thirst—no pain in any part. His age is 47, and he has spent his life in agricultural pursuits, and temperate habits. Always enjoyed good health till the present illness, which is of about two months duration. Tonics, mild nutrition, and wine, were ordered. In a fortnight, he felt better, but there was no change in his complexion. In September, and occasionally afterwards, Drs. Kellie and R. Hamilton saw the patient with our author; but they could not solve the nature of the complaint. After trying a short sea voyage, and drinking of a chalybeate spring, he returned, in the middle of October, with a loss of flesh and strength, his legs swollen, his skin still exanguious, with great debility. During the two succeeding months, the disease preserved a uniformity of symptoms, but gradually increasing in degree. About the middle of January, 1822, the œdema had become general, to which was added an evident effusion in the chest. He died in a few weeks after this, under symptoms of hydrothorax.

*Dissection.* Externally, the colour was nearly the same as has been described during life. Not a drop of blood escaped on dividing the scalp—dura mater pale, and presenting but few vessels, which were empty. "Near the vertex, and to the left of the sinus, was a considerable ossification imbedded in the plicæ of the membrane; it was an inch long, rough, and irregular." The pia mater was pale, its vessels containing a pale serum, and a considerable quantity of air; a slight effusion under the arachnoid coat. The substance of the brain was very soft and pultaceous, presenting very few vessels; and there was very little distinction of colour between the medullary and cineritious portions. The ventricles contained about two drachms of serum, and about two ounces were found at the basis. The lateral sinuses were moderately filled with pale fluid blood—the arteries at the basis empty. In the thorax, about three pounds of a lemon-coloured serum were effused—the lungs of a pale grey colour, without any signs of gravitated blood. Pericardium contained about an ounce of serum—heart pale, like flesh macerated many days in water. The inner coat of the aorta was of a fine red colour for some inches, but without any turgescence or ossification. There was no exudation of blood, on cutting into the abdominal viscera; and the spleen was the only viscus which preserved its usual colour. The muscles, throughout the body, were pale like the heart. The arteries were universally empty, as were the jugular, humeral, and femoral veins. The vena cava, about the bifurcation, contained a little blood.

We cannot expect to have any light thrown on the subject of Anæmia, until a most minute scrutiny be made of the state of the digestive organs, thoracic duct, and lungs, in subjects who die of the disease. This can only be done in hospital practice, or in an anatomical theatre.

### Art. III.

*Case of Hydrocephalus, with bifid Brain.* By. Andrew Duncan, jun. M. D. &c. &c.

This is a case of extreme rarity, and consequently of proportionate curiosity. In our author's researches (and they have been extensive enough) he has met with no case of bifid brain in a child that survived birth. The infant in question, was a female, and survived seven months, having been seen by Dr. Duncan, and several other medical gentlemen, occasionally. For some time before death, the greatest circumference of the head, was 29 inches. All the functions appeared to be natural; but the countenance, and other parts of the body, were emaciated. The child could never sit up, on account of the weight of the head—yet she was sometimes lively, and evidently received pleasure from being spoken to, or played with. She died on the 1st October, and the head was minutely examined, by the late Dr. Gordon—a sufficient voucher for the accuracy of the dissection.

The dimensions were 28½ inches in circumference—and 16½ over the vertex, from ear to ear. We shall be able to give but very few of the particulars of this examination, which, to many people, will appear needlessly minute.

The dura mater adhered closely to the bones, wherever they existed—and where they were defective, it adhered to the inner surface of the integuments. It was healthy. The water was drawn off by a puncture, through the most prominent point of the occiput. The quantity was 136 ounces, by weight; transparent, and colourless like spring water. The surfaces of the two hemispheres were separated about 4 inches, except at their anterior extremities. The corpus callosum was wholly wanting, except two white bands, as were the fornix, septum lucidum, and anterior commissure. The ventricles were considerably enlarged, and appeared to form one common cavity with the parietes of the cranium for the reception of the water. The further description, occupying many pages, must be perused by the curious in the original. The following passage will be read with some interest.

"The case occurred soon after Dr. Gordon and Dr. Spurzheim had published their respective opinions regarding the pathological state of the brain in chronic hydrocephalus, and we proceeded to the examination with the greatest anxiety, fully expecting that the facts would bear decidedly upon the chief point at issue, whether the brain was merely distended and unfolded, as maintained by Spurzheim, or, whether the enlargement of the ventricles was owing to interstitial absorption, according to the opinion of Dr. Gordon. We never doubted that the water would be found within the ventricles. But in this respect we were completely disappointed, for there was neither absorption of the brain, nor distension of the ventricles. The whole brain, cerebrum and cerebellum, weighed 8040 grains, of which the cerebellum was 1200, leaving 6840 for the brain proper, independently of the dura mater, and what Dr. Gordon has called the new membrane, but which I am disposed to consider as the arachnoid coat thickened." 219.

Dr. Duncan has not been able to ascertain the average weight of the brain and cerebellum in infants of seven months of age; but from a table of weights of brains, given by the brothers Weuzel, he conjectures that there was no absorption of brain in the case here related—"and no distention of the cavities of the ventricles." We know not how to reconcile this statement with that of Dr. Gordon himself, at page 212, where he distinctly avers that "the ventricles were considerably enlarged."* Be this as it may, the case does not appear to bear upon the point at issue between Drs. Spurzheim and Gordon, excepting so far as it proved that the power by which the fluid was effused, and which was capable of distending the dura mater, skull, and its integuments, so as to contain nine times its natural volume of contents, did not cause any appreciable absorption of the cerebral substance.

Our author doubts (and with reason) the existence of what authors call external hydrocephalus. We have never seen an instance of it, nor any one that did. In many cases of chronic hydrocephalus internus, as Dr. D. justly observes, the ventricles are so distended, and their parietes so attenuated, that

* Although the ventricles made common parietes with the whole cavity in which the water was contained, it can hardly, we think, be said that they did not suffer *distention*, as well as enlargement.—REV.

the head becomes translucent as a hydrocele, and the hemispheres form a mere membranous bag, which has often been ruptured in the act of opening the head. The water is then supposed to lie in direct contact with the membranes, and between them and the brain. But this is evidently an error.

For many ingenious physiological and pathological observations on the origin of mal-formations in general, we must refer to the original and very able paper itself.

### Art. IV.

*A Case of Phrenitis, with great cerebral Congestion, successfully treated, in India, by opening the Radial Artery.* By J. P. Rhind, Esq. Surgeon of Cavalry.

This is a case of a very extraordinary kind—or rather, it gave rise to extraordinary conceptions in the mind of the medical attendant, and was treated in a most extraordinary manner. Mr. Rhind was called, at midnight, to his native assistant surgeon, who had been "*taken suddenly and dangerously ill.*" He found him in a low muttering delirium, pulse quick, hard, and small—"eyes (query the pupils) much dilated"—lying sometimes quiet for a few seconds, breathing labouriously—then starting up, and vociferously calling on those around him, unconscious of their presence—then again muttering prayers, &c. There was no suspicion of intoxication. While sending off for his lancets, Mr. R. found the pulse sink rapidly, and when a vein was opened, no blood would flow. The same was the case with the jugular vein. The temporal artery being cut, a very feeble stream of blood came forth. The extremities became cold—"and neither his breathing nor the pulsation of his heart was perceptible." "He was, to all appearance, dead." Mr. Rhind, in order to resuscitate his assistant, laid open the radial artery with a scalpel; "but only a few drops of blood oozed out." Presently, however, the blood came in an uninterrupted stream, which gradually enlarged, and, at length, came *per saltum*, when the native doctor opened his eyes, and after heaving a deep sigh, observed that he was better. He was put to bed, and a slight tendency to relapse being evinced, some more blood was permitted to flow, and presently the doctor was well.

As failure does not necessarily prove mal-treatment, neither does success always prove sound judgment, in the *methodus medendi.* Mr. Rhind being at too great a distance, we beg leave to ask Dr. Barclay, who communicates this paper, and whose extensive experience at the bed side must long ago have rendered him familiar with the phenomena of disease, whether the above complaint of the native doctor presented the symptoms of phrenitis?—To our apprehension, there is not the shadow of a proof of inflammation of the brain. Such disturbance of the sensorial functions and derangement of the heart's action, we have often, indeed, seen from disordered stomach, and noxious ingesta—yet these complaints are very different from phrenitis. But, waiving all question about the nature of the disease, we again ask Dr. Barclay, whether he approves of cutting the radial artery of a man whose extremities are cold, whose breathing has ceased, and whose heart no longer pulsated?—Does he conceive that the mere cutting of the radial artery in the above state of syncope, had any thing to do with the resuscitation from apparent death—or the re-excitement of the heart and arteries? The fact is, that the powers of life returned—blood flowed—and the patient got well; but we do aver that the pathology of the disease was misunderstood—and that the remedy was misapplied, in respect to time at least, whatever was the nature of the complaint.

### ART. V.

*Case of Dysphagia, with Abscess involving the Œsophagus, Trachea, and Lungs.* By DAVID HAY, M.D.

In December 1821, a gentleman, aged 54 years, and who had generally enjoyed good health, began to suffer from symptoms of dyspepsia, such as loss of appetite, flatulence, heart-burn, pain after eating, disposition to spit frothy mucus, with costive bowels, and furred tongue. In the succeeding month, he had a feeling of obstruction in swallowing his food, which he attributed to flatulence. In February, March, and April, 1823, he was better, and able to attend to his business. In May, the symptoms became aggravated, the difficulty of swallowing some kinds of food being greater than of others. He now began to complain of pain across the upper part of the right breast—he was sometimes sick, and brought up ropy mucus. A probang was passed into the œsophagus, without difficulty, but occasioned pain at the pit of the stomach. Having gone into the country, he returned in the latter end of July, having lost flesh and strength. His complexion was now sallow, his pulse frequent, bowels constipated. In the beginning of August, he had a feverish attack, with pain in the right breast, for which he was bled, blistered, and purged. About the 6th, he began to cough, particularly on taking food, and at night. On the 24th, he had a rigor, followed by fever—the pain, dysphagia, and cough troublesome. On the 26th, there was complete obstruction in the gullet, and an elastic tube was stopped opposite the upper part of the sternum. A gum-catheter, however, was passed into the stomach. Through this tube nothing could be got into the organ, without being instantly thrown back, threatening suffocation, and inducing violent coughing. He now began to expectorate large quantities of a sero-purulent fluid, having a most offensive smell. He sunk on the 1st September.

*Dissection.* There was a large abscess in the upper and posterior part of the right lung, comprehending in its parietes the œsophagus and trachea. The œsophagus was destroyed by ulceration, to the extent of half its circumference, and upwards of four inches in its length. An opening had taken place into the trachea, tracing the œsophagus downwards, and on laying it open, a line of tubercles, somewhat larger than split peas, and of firm texture, was seen lying immediately behind, or in the substance of the mucous membrane, which, in some places, was slightly abraded, or ulcerated. This line of tubercles led to a larger one, nearly the size of an almond, placed on the exterior surface of the œsophagus, near the cardia. A hard mass of enlarged lymphatic glands was found in the angle betwixt the œsophagus and stomach, which must have compressed the opening into the latter. The stomach itself was sound.

### Art. VI.

### *Case of Malformation of the Heart.* By W. F. Holmes, M.D.

The varieties of malformation in the heart are very numerous, and the one related here, though perhaps unique in its appearance, produced effects similar to many others recorded by authors.

The patient reached the age of 21 years, and would probably have lived many years longer, had he led a regular life. He suffered much, however, from palpitation, attended by a peculiar blueness of the cheeks and lips, more remarkable at one time than at another. The palpitation was aggravated by motion, and the dyspnœa, sometimes threatened suffocation. At length dropsy came on, and the patient died. On dissection, the right auricle was found so enlarged as to hold a pint—and the foramen ovale pervious, admitting the point of the little finger. There was also a communication between the right auricle and left ventricle—and no opening between the two right chambers themselves. The right ventricle was very small, and communicated with the left by an opening in the septum ventriculorum, furnished with a valvular apparatus. The course of the circulation, in this case, must have been as follows:—when the auricles acted, the blood was thrown from the right auricle not only into the *left* ventricle, but from that, in part, into the right ventricle, through the opening abovementioned, and from thence it was thrown into the pulmonary artery and so on. There was thus a mixture of black and red blood in the left chambers of the heart, and an imperfect aeration of this fluid in the general circulation.

### ART. VII.

*Case of Tubercular Disease of the Peritoneum and Omentum, combined with Tympanitic Affection.* By WILLIAM MONCRIEF, M.D. &c.

A widow woman, 40 years of age, applied to the New Town Dispensary, on the 5th October, 1821, complaining of loss of appetite, indigestion, constipation, globus hystericus, foul tongue, &c. She said her ailments commenced only three weeks previously, after exposure to wet and cold. On the 10th, when visited at home, she was found with nausea and vomiting, constipation, pain all over the abdomen, but severer in the right hypochondrium and left ilium. The abdomen was very tense and tympanitic; no alvine evacuations for four days. She dragged out a miserable existence till the 31st of the same month, when death put a period to her sufferings.

*Dissection.* On opening the abdomen, four English pints of serum flowed from it. The omentum appeared reddish and tuberculated, extending over nearly all the abdomen, and firmly attached to the fundus uteri. When turned back, it was found much thickened—in several places as much as half an inch. Both its sides, and also the peritoneum lining the abdomen and covering the intestines, were thickly studded with tubercles. The small intestines adhered to each other, and were slightly covered with a purulent secretion. The covering of the liver was tuberculated, but its substance was healthy. There was scirrhus of the pylorus.

It is evident that the tubercular affection was going on for a much longer time than the patient stated—it was only when an inflammatory condition obtained that she was rendered incapable of pursuing her avocations.

Here we must close our second analytical article. We shall return to this volume in our next number.

